# Is It Necessary to Remove the Implants After Fixation of Thoracolumbar and Lumbar Burst Fractures Without Fusion? A Retrospective Cohort Study of Elderly Patients

**DOI:** 10.3389/fsurg.2022.921678

**Published:** 2022-07-04

**Authors:** Xiangyu Xu, Yuan Cao, JiXing Fan, Yang Lv, Fang Zhou, Yun Tian, Hongquan Ji, Zhishan Zhang, Yan Guo, Zhongwei Yang, Guojin Hou

**Affiliations:** ^1^ Department of Orthopedics, Peking University Third Hospital, Beijing, China; ^2^ Engineering Research Center of Bone and Joint Precision Medicine, Ministry of Education, Beijing, China

**Keywords:** thoracolumbar and lumbar, burst fractures, implant removal, non-fusion fixation, elderly patients

## Abstract

**Objective:**

Fractures of the thoracolumbar spine are the most common fractures of the spinal column. This retrospective cohort study aimed to determine whether it is necessary to remove implants of patients aged over 65 years after the fixation of thoracolumbar and lumbar burst fractures without fusion.

**Methods:**

This retrospective cohort study included 107 consecutive patients aged ≥65 years without neurological deficits, who underwent non-fusion short posterior segmental fixation for thoracolumbar or lumbar burst fractures. Outcome measures included the visual analog score (VAS), Oswestry Disability Index (ODI), residual symptoms, complications, and imaging parameters. Patients were divided into groups A (underwent implant removal) and B (implant retention) and were examined clinically at 1, 3, 6, and 12 months postoperatively and annually thereafter, with a final follow-up at 48.5 months.

**Results:**

Overall, 96 patients with a mean age of 69.4 (range, 65–77) years were analyzed. At the latest follow-up, no significant differences were observed in functional outcomes and radiological parameters between both groups, except in the local motion range (LMR) (*P* = 0.006). Similarly, between preimplant removal and the latest follow-up in group A, significant differences were found only in LMR (*P* < 0.001). Two patients experienced screw breakage without clinical symptoms. Significant differences were only found in operation time, blood loss, ODI, and fracture type between minimally invasive group and open group.

**Conclusions:**

Similar radiological and functional outcomes were observed in elderly patients, regardless of implant removal. Implant removal may not be necessary after weighing the risks and benefits for elderly patients. Patients should be informed about the possibility of implant breakage and accelerating degeneration of adjacent segments in advance.

## Introduction

Fractures of the thoracolumbar spine are the most common fractures of the spinal column (1). These injuries can lead to problems, such as paraplegia, pain, bowel and bladder dysfunction, and sexual dysfunction (1, 2). Stabilization with pedicle screws is a prevalent treatment for thoracolumbar and lumbar fractures without neurological deficit (3). The operation can establish stability and restore spinal alignment to prevent neurologic deterioration and alleviate pain (4, 5).

Surgical site infection, pain, implant breakage or loosening, and soft tissue irritation are indications for implant removal (6, 7). However, in some successful asymptomatic cases, the indications for implant removal remain controversial. Pedicle screw removal may eliminate the potential risks of metal fretting, infection, micromotion, disc degeneration, allergic reaction, and osteopenia caused by stress shielding (5, 7, 8); however, implant removal as a second surgical operation is accompanied by the risks of surgical site infection, neurovascular injury, and refracture (7, 9).

To date, most published studies have only discussed implant removal because of symptoms associated with implants (8, 10). Jeon et al. (7) reported that in patients treated successfully for thoracolumbar burst fractures, pedicle screw removal after surgery was beneficial because it alleviated pain and disability. However, Chou et al. (11) found no significant differences in the treatment outcomes between patients in whom the implants were removed, and those in whom they were not removed after fixation of burst fractures of the thoracolumbar and lumbar spine. Moreover, all these studies discussed young cases (mean age ≤45 years), and few studies have focused on elderly patients with thoracolumbar and lumbar fractures. The primary goal of this study was to determine whether it is necessary to remove the implants of patients that were >65 years of age after fixation of thoracolumbar and lumbar spinal fractures without fusion.

## Materials and Methods

This was a retrospective cohort study. A total of 107 consecutive patients aged ≥65 years without neurological deficits, who underwent non-fusion short posterior segmental fixation using pedicle screws for thoracolumbar or lumbar burst fractures (type A3 or A4 in terms of AO spine classification) at our university hospital between August 2011 and August 2018 were included in the study. Of these, 11 patients were lost to follow-up because they did not finish the follow-up protocol or could not be contacted. The study was approved by the regional ethics committee (IRB00006761-M2020579), and the need for informed consent was waived by the ethics committee.

We excluded patients with neurological deficits, severe injury of the facet joints, spine malformation, polytrauma, and other situations that affected patients' waist and lower limb functions or bone healing (e.g., autoimmune disease, ankylosing spondylitis, and systemic corticosteroid treatment).

Fracture fixation was performed at an average of 4.8 days after injury. The operation used a four-transpedicle-screw fixation without fusion, ranging one level above and below the fracture site. In 64 patients, percutaneous pedicle screws were used, while in the remaining 32 patients for whom satisfied closed reduction was not achievable on the operation table, a posterior midline approach using Schanz screws was employed. Ambulation was initiated within 2 days after surgery with the protection of a thoracolumbosacral orthosis brace for 6–8 weeks. We recommend a postoperative brace for elderly patients in order to further increase local stability, relieve pain, and encourage early rehabilitation exercises. In our department, implant removal was considered at least 12 months after surgery when fracture healing was observed, or when irritating symptom from internal fixation occurred. Some surgeons recommended implant removal to their patients, while others did not recommend implant removal for elderly patients. Therefore, the patients were divided into two groups retrospectively: group A (*n* = 46), whose implants were removed, and group B (*n* = 50), whose implants were retained.

The patients were examined clinically at 1, 3, 6, and 12 months postoperatively, and annually thereafter, with a final mean follow-up duration of 50.2 (range, 36–102) months in group A and 46.9 (range, 24–96) months in group B. In our study, when a blurred fracture line or increased density and callus formation of the fractured vertebrae were visualized on the X-ray, the bony union of the vertebrae was confirmed. All implants were removed in group A at a mean of 16.8 (range, 12–34) months. Plain radiographs (anteroposterior and lateral views) and the visual analog score (VAS) for back pain were obtained preoperatively and postoperatively at 1, 3, 6, and 12 months, and at the final follow-up. Preoperative computed tomography and magnetic resonance images of the spine were also obtained. Preimplant removal and final follow-up flexion and extension lateral radiographs were obtained for all patients and analyzed. We considered that there was no bone fusion when patients lack a clear continuous callus and local vertebral body movement was observed on the last flexion and extension lateral radiographs. Function was assessed using the Oswestry Disability Index (ODI), which was obtained at 1, 3, 6, and 12 months and at the final follow-up.

**Figure 1:**
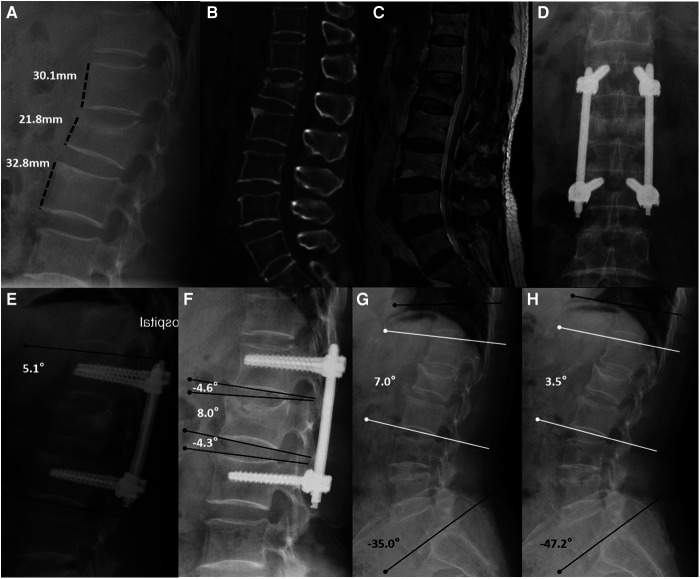
Non-fusion fixation using percutaneous pedicle screws for L1 fracture (type A3). A 67-year-old woman from group A presented in 2017 with lower back pain without neurological deficit after a fall. (**A**–**C**) Lateral radiography, sagittal computed tomography, and T-2 weighted magnetic resonance imaging were taken preoperatively. Anterior vertebral body-to-height ratio = 21.8/[(30.1 + 32.8)/2] × 100% = 69.3%. (**D**,**E**) At the 1-month follow-up, anteroposterior and lateral radiographs show that the L1 vertebral body height had recovered, and the kyphotic angle had decreased (Cobb A = 5.1°). (**F**) At the 1-year follow-up, lateral radiographs were taken before implant removal. Cobb B = 8.0° and Cobb C = (−4.6°) + (−4.3°) = −8.9°. (**G**,**H**) At the 40-month follow-up, flexion and extension lateral radiographs show the motion range of the spine. Local motion range = 7.0°–3.5° = 3.5° and total motion range = (−35.0°) − (−47.2°) = 12.2°.

All imaging parameters, such as kyphotic angles and anterior vertebral body height, were measured on standing lateral radiographs, and all angles were measured using Cobb's technique (kyphosis defined as positive value and lordosis defined as negative values). The anterior vertebral body-to-height ratio (AVHR) (12) was used to determine the change in the injured vertebral body height (Figure 1A). The angle between the upper endplate and lower endplate of a fixed segment was defined as Cobb A, and the angle between the upper and lower endplates of the injured vertebra was defined as Cobb B. Cobb C was defined as the sum of the Cobb angles of the discs just above and below the injured vertebra (Figures 1E,F). In preimplant removal and final follow-up radiographs, to determine the motion range of fixed segments and total thoracolumbar and lumbar segments in flexion and extension lateral radiographs, we used the local motion range (LMR) and the total motion range (TMR). The LMR was calculated by subtracting the value of Cobb A in the extension radiograph from the value of Cobb A in the flexion radiograph. The TMR was based on the differences in the kyphotic angles of the lower endplate of T10 to the lower endplate of L5 between dynamic flexion-extension lateral radiographs (Figures 1G,H). All measurements were performed by two authors (X-XY and C-Y) and were obtained three times using the arithmetic mean.

Statistical analyses were performed using the Statistical Package for the Social Sciences for Windows (version 25.0; IBM Corp, Chicago, Illinois). Data are reported as mean ± standard error of the mean for continuous data, and as frequencies (percentages) for categorical variables, unless otherwise noted. The Student's *t*-test (for continuous data), Chi-squared test, and Fisher's exact test (for categorical data) were used to evaluate the parameters between the two groups. Statistical significance was set at *P* ≤ 0.05.

## Results

A total of 96 patients (96/107, 89.7%) with a mean age of 69.4 (range, 65–77) years were finally analyzed. The demographics, mechanism of burst fractures, injury level, surgical approach, follow-up time, and fracture type of AO spine classification are shown in Table 1. No significant differences were found between groups A and B.

**Table 1:** Patient characteristics at the time of injury.

	Total (*n* = 96)	Group A (*n* = 46)	Group B (*n* = 50)	*P*-Value
Gender				0.556
Male	45 (46.9)	23 (50.0)	22 (44.0)	
Female	51 (53.1)	23 (50.0)	28 (56.0)	
Age (years)	69.4 ± 2.2	69.8 ± 2.5	69.1 ± 1.9	0.120
Body mass index	25.1 ± 3.1	25.2 ± 2.8	24.9 ± 3.4	0.686
Injured level				0.079
T11	2 (2.1)	0 (0)	2 (4.0)	
T12	16 (16.7)	6 (13.0)	10 (20.0)	
L1	46 (47.9)	21 (45.7)	25 (50.0)	
L2	21 (21.9)	10 (21.7)	11 (22.0)	
L3	6 (6.2)	6 (13.0)	0 (0)	
L4	5 (5.2)	3 (6.6)	2 (4.0)	
Injury mechanism				0.555
Fall	57 (59.4)	27 (58.7)	30 (60.0)	
Traffic accident	13 (13.5)	6 (13.0)	7 (14.0)	
Fall from a height	21 (21.9)	9 (19.6)	12 (24.0)	
Others	5 (5.2)	4 (8.7)	1 (2.0)	
Fracture type				0.827
A	87 (90.6)	42 (91.3)	45 (90.0)	
B	9 (9.4)	4 (8.7)	5 (10.0)	
Follow-up time (mths)	48.5 ± 22.4	50.2 ± 25.9	46.9 ± 19.6	0.302
Surgical approach				0.312
Percutaneous	64 (66.7)	33 (71.7)	31 (62.0)	
Open	32 (33.3)	13 (28.3)	19 (38.0)	

**Figure 2:**
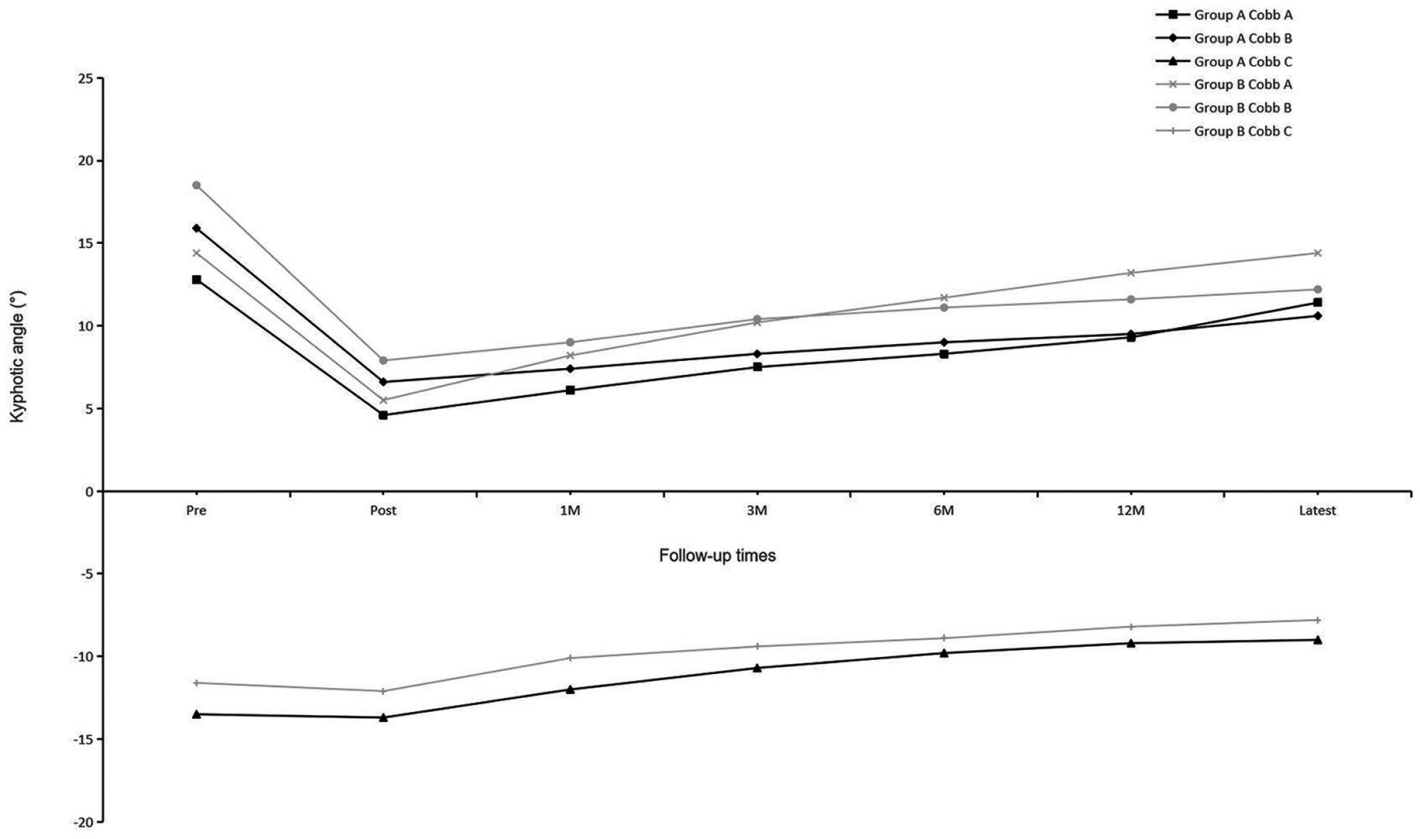
Mean kyphotic angles (Cobb A, Cobb B, and Cobb C) of the two groups at each time point.

**Figure 3:**
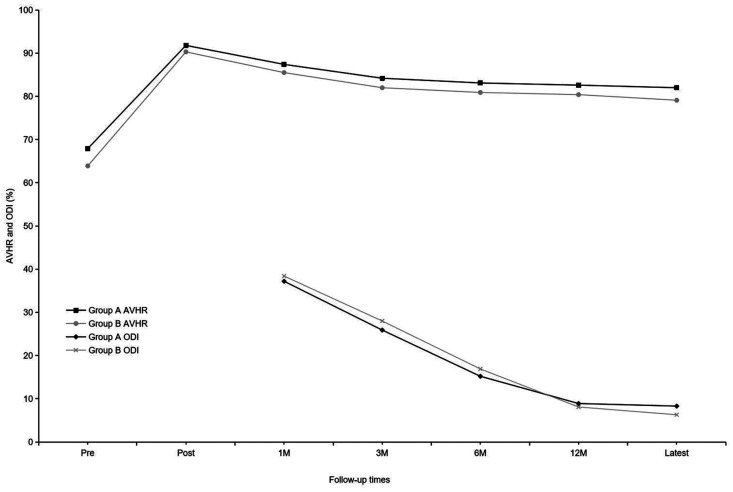
Mean anterior vertebral body-to-height ratio and mean Oswestry Disability Index of the two groups at each time point.

Before implant removal, functional outcomes and radiological parameters, such as Cobb A, Cobb B, and Cobb C (Figure 2), VAS, AVHR, and ODI (Figure 3), were collected. No significant differences were found in these data between the two groups within the 1-year follow-up period (Table 2).

**Table 2:** Clinical and radiological parameters before implant removal.

	Total (*n* = 96)	Group A (*n* = 46)	Group B (*n* = 50)	*P*-Value
AVHR (%)				
Pre-operative	66.0 ± 9.8	67.9 ± 10.3	63.9 ± 8.8	0.124
Post-operative	91.1 ± 5.7	91.8 ± 5.6	90.3 ± 5.8	0.306
1 month	86.4 ± 7.4	87.4 ± 6.7	85.5 ± 8.0	0.335
3 months	83.0 ± 8.6	84.2 ± 7.0	82.0 ± 10.0	0.343
6 months	82.0 ± 8.6	83.1 ± 7.2	80.9 ± 9.6	0.331
12 months	81.5 ± 8.5	82.6 ± 7.5	80.4 ± 9.4	0.323
Cobb A (°)				
Pre-operative	13.7 ± 13.1	12.8 ± 15.9	14.4 ± 10.2	0.651
Post-operative	5.1 ± 12.4	4.6 ± 14.0	5.5 ± 10.9	0.794
1 month	7.2 ± 12.2	6.1 ± 14.0	8.2 ± 10.4	0.528
3 months	8.9 ± 12.4	7.5 ± 14.3	10.2 ± 10.5	0.410
6 months	10.1 ± 12.3	8.3 ± 13.7	11.7 ± 10.8	0.301
12 months	11.3 ± 12.2	9.3 ± 13.9	13.2 ± 10.4	0.240
Cobb B (°)				
Pre-operative	17.2 ± 7.1	15.9 ± 7.7	18.5 ± 6.5	0.175
Post-operative	7.3 ± 4.1	6.6 ± 3.9	7.9 ± 4.3	0.244
1 month	8.2 ± 4.3	7.4 ± 4.0	9.0 ± 4.4	0.172
3 months	9.4 ± 4.5	8.3 ± 4.6	10.4 ± 4.4	0.096
6 months	10.1 ± 4.6	9.0 ± 4.6	11.1 ± 4.5	0.088
12 months	10.5 ± 4.7	9.5 ± 4.7	11.6 ± 4.5	0.092
Cobb C (°)				
Pre-operative	−12.5 ± 5.4	−13.5 ± 5.0	−11.6 ± 5.6	0.177
Post-operative	−12.9 ± 4.1	−13.7 ± 4.5	−12.1 ± 3.5	0.157
1 month	−11.0 ± 4.9	−12.0 ± 4.7	−10.1 ± 4.9	0.145
3 months	−10.0 ± 4.8	−10.7 ± 4.6	−9.4 ± 4.9	0.300
6 months	−9.3 ± 4.6	−9.8 ± 4.3	−8.9 ± 4.9	0.499
12 months	−8.7 ± 4.4	−9.2 ± 4.0	−8.2 ± 4.7	0.354
VAS for back pain				
Pre-operative	6.8 ± 1.1	6.7 ± 1.1	6.9 ± 1.1	0.518
Post-operative	2.8 ± 1.1	2.5 ± 1.2	3.0 ± 1.0	0.073
1 month	2.5 ± 1.2	2.3 ± 1.3	2.7 ± 1.1	0.264
3 months	2.0 ± 1.7	1.7 ± 1.7	2.2 ± 1.6	0.267
6 months	1.2 ± 1.3	1.2 ± 1.1	1.2 ± 1.4	0.869
12 months	0.9 ± 1.3	1.0 ± 1.1	0.8 ± 1.4	0.481
ODI (%)				
1 month	37.8 ± 20.9	37.2 ± 23.4	38.4 ± 18.7	0.825
3 months	27.0 ± 20.3	25.9 ± 21.6	28.0 ± 19.4	0.706
6 months	16.1 ± 15.5	15.2 ± 15.5	16.9 ± 15.7	0.681
12 months	8.5 ± 11.3	8.9 ± 10.8	8.1 ± 11.9	0.788

*AVHR, anterior vertebral body-to-height ratio; ODI, Oswestry Disability Index; VAS, visual analogue score.*

For residual symptoms, chronic back pain was defined by a VAS score of ≥3 at the latest follow-up, and subjective lumbar movement restriction was defined when patients specifically complained of restricted motion of the lumbar spine at the latest follow-up. No significant differences were observed in the functional outcomes and radiological parameters (Table 3) between the two groups, except for LMR (*P* = 0.006) at the latest follow-up. Comparison of preimplant removal and the latest follow-up in group A (Table 4) revealed significant differences only in LMR (*P* < 0.001).

**Table 3:** Radiological and functional outcomes at the latest follow-up.

	Total (*n* = 96)	Group A (*n* = 46)	Group B (*n* = 50)	*P*-Value
AVHR (%)	80.5 ± 9.0	82.0 ± 8.6	79.1 ± 9.3	0.231
Cobb A (°)	12.9 ± 12.3	11.4 ± 14.4	14.4 ± 10.1	0.367
Cobb B (°)	11.4 ± 4.8	10.6 ± 5.2	12.2 ± 4.4	0.216
Cobb C (°)	−8.4 ± 4.6	−9.0 ± 4.6	−7.8 ± 4.6	0.327
LMR (°)	4.0 ± 1.3	4.4 ± 1.4	3.6 ± 1.0	0.019*
TMR (°)	10.5 ± 3.9	11.1 ± 4.7	9.9 ± 2.8	0.252
VAS for back pain	1.0 ± 1.5	1.2 ± 1.6	0.8 ± 1.4	0.289
ODI (%)	7.3 ± 11.1	8.3 ± 11.0	6.3 ± 11.2	0.502
Screw breakage	2 (2.1)	0 (0)	2 (4.0)	0.496
SLMR	3 (3.1)	0 (0)	3 (6.0)	0.243
Chronic back pain	11 (11.5)	6 (13.0)	5 (10.0)	0.640

*Statistically significant P-values were the results after comparison between the two groups.

*AVHR, anterior vertebral body-to-height ratio; ODI, Oswestry Disability Index; VAS, visual analogue score; LMR, local motion range; TMR, total motion range; SLMR, subjective lumbar movement restriction.*

**Table 4:** Results comparison of group A between preimplant removal and the latest follow-up.

	Preimplant removal	Latest follow-up	*P*-Value
AVHR (%)	84.3 ± 7.4	82.0 ± 8.6	0.308
Cobb A (°)	9.6 ± 14.1	11.4 ± 14.4	0.653
Cobb B (°)	9.6 ± 4.7	10.6 ± 5.2	0.460
Cobb C (°)	−11.1 ± 5.4	−9.0 ± 4.6	0.130
LMR (°)	3.1 ± 1.0	4.4 ± 1.4	<0.001*
TMR (°)	10.1 ± 4.6	11.1 ± 4.7	0.444
VAS for back pain	1.1 ± 1.4	1.2 ± 1.6	0.785
ODI (%)	8.7 ± 10.7	8.3 ± 11.0	0.907

*Statistically significant P-values were the results after comparison between the two groups.

*AVHR, anterior vertebral body-to-height ratio; ODI, Oswestry Disability Index; VAS, visual analogue score; LMR, local motion range; TMR, total motion range.*

**Figure 4:**
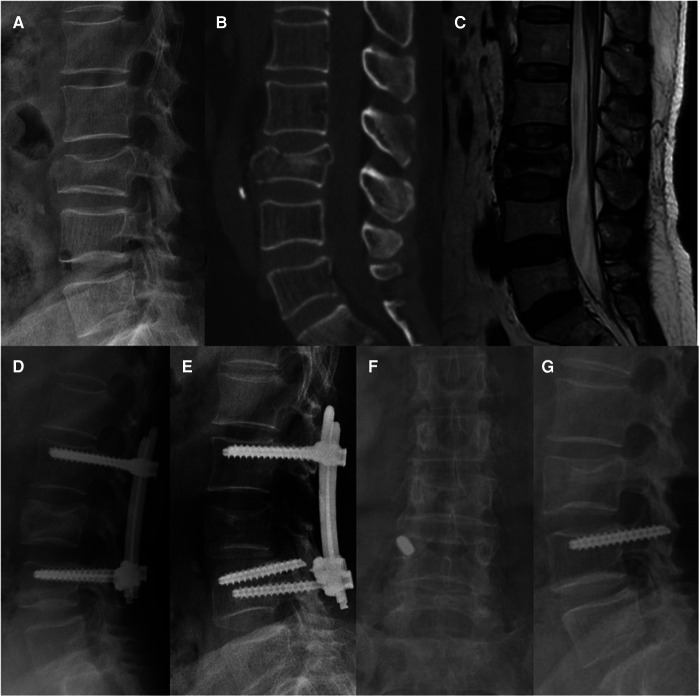
Non-fusion fixation using percutaneous pedicle screws for L3 fracture (type A4). A 68-year-old man from group B presented in 2016 with severe lower back pain without neurological deficit after a fall. (**A–C**) Preoperative lateral radiography, sagittal computed tomography, and T-2 weighted magnetic resonance imaging were taken 6 h after the fall. (**D**) Lateral radiography was taken at the 3-month follow-up. (**E**) At the 21-month follow-up, screw breakage was found without any symptoms. Implant removal surgery was subsequently performed, the broken screw could not be removed. (**F**,**G**) At the 56-month follow-up, anteroposterior and lateral radiographs show that the L3 vertebral body height and kyphotic angle were almost maintained, and the broken screw was still in the right pedicle of L4.

Two patients experienced screw breakage without clinical symptoms and obvious cause 15 and 21 months after surgery, respectively. Consequently, they accepted implant removal surgery (Figure 4). No screw or rod breakage was found in the other patients before implant removal. No neurological deficit, spondylolisthesis, or bony fusion were observed in either group at the latest follow-up.

**Figure 5:**
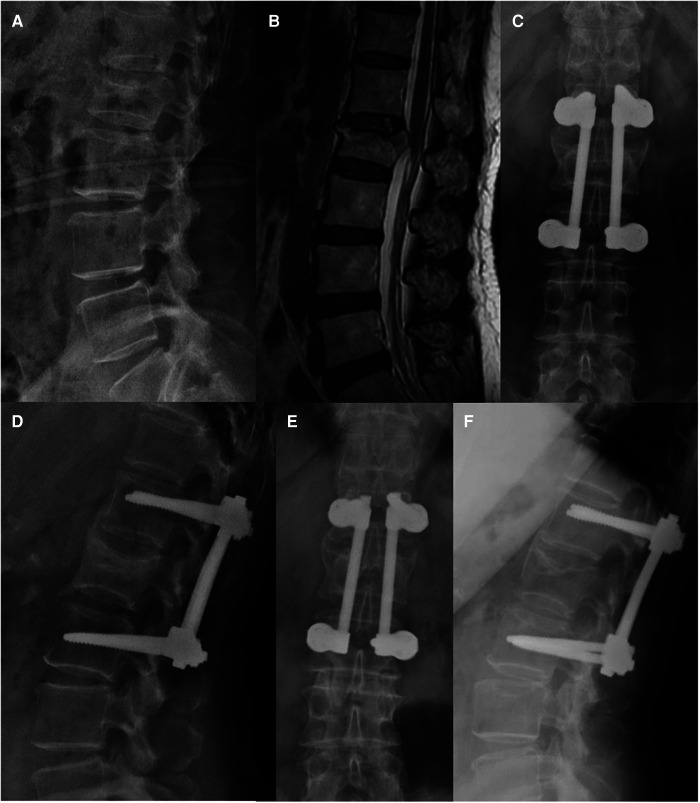
Non-fusion short-segment fixation using Schanz screws for L2 fracture (type A3). A 66-year-old woman from group B presented in 2014 with severe lower back pain without neurological deficit after a traffic accident. (**A**,**B**) Preoperative lateral radiography and T-2 weighted magnetic resonance imaging show a posterior vertebral wall fracture of L2. (**C**,**D**) Anteroposterior and lateral radiographs at the 1-year follow-up. (**E**,**F**) At the 51-month follow-up visit, anteroposterior and lateral radiographs show that the implant position remains unchanged.

In our study, 64 patients underwent percutaneous pedicle screw fixation (minimally invasive group) and in 32 patients Schanz screws were used (Figure 5) with the posterior midline approach (open surgery group). Statistically significant differences were found in operation time, blood loss, ODI (1, 3, and 6 months), and fracture type between the two groups (Table 5). No significant differences were found in the other functional and radiological outcomes between the two groups.

**Table 5:** Comparison of two surgical approaches at the latest follow-up.

	Minimally invasive (*n* = 64)	Open (*n* = 32)	*P*-Value
Operation time (min)	84.4 ± 22.5	97.9 ± 20.4	0.005*
Blood loss (ml)	60.9 ± 50.9	152.8 ± 83.6	<0.001*
Hospitalization time (day)	5.6 ± 2.9	6.0 ± 1.8	0.357
Screw breakage	2 (3.1)	0 (0)	0.551
SLMR	2 (3.1)	1 (3.1)	1.000
Chronic pain	8 (12.5)	3 (9.4)	0.747
ODI (%)			
1 month	28.7 ± 14.8	41.8 ± 22.0	0.012*
3 months	19.4 ± 15.8	30.3 ± 21.4	0.039*
6 months	10.7 ± 10.1	18.5 ± 16.9	0.038*
12 months	5.7 ± 9.0	9.7 ± 12.1	0.237
Latest follow-up	5.9 ± 10.2	7.9 ± 11.5	0.559
Fracture type			0.006*
A	62 (96.9)	25 (78.1)	
B	2 (3.1)	7 (21.9)	

*Statistically significant P-values were the results after comparison between the two groups.

*SLMR, subjective lumbar movement; ODI, Oswestry Disability Index.*

## Discussion

For thoracolumbar and lumbar compression fractures without neurological deficit, stabilization with pedicle screws and percutaneous kyphoplasty (PKP) are both safe and effective in elderly patients. However, some complications, such as bone cement leakage and adjacent vertebral fractures, may occur when PKP is used to treat burst fractures (13, 14). In our study, all burst fractures were caused by moderate-to-high violence trauma. Therefore, we did not choose PKP for these patients. And we did not use bone cement for screw augmentation either, because there is a remaining risks of cement extravasation into the venous system, spinal canal, or disk space (15) and most patients with osteoporosis after pedicle screw fixation have a good prognosis in our experience. Non-operative treatment for burst fractures without neurological deficit has been reported to yield good results, although a great residual kyphotic angle has been noted (16, 17). Therefore, we recommend surgical treatment for patients who can tolerate surgery after preoperative evaluation, especially for elderly patients who might have a higher risk of long-term bed rest.

Screw breakage and progressive kyphosis are common complications of spinal fixation surgery, even in surgeries with successful fusion (16). However, many studies have reported that functional outcomes were not affected by implant failure (16, 18, 19). Although solid fusion was achieved, the incidence of implant failure ranged from 9.5 to 20% (18, 19). Chou et al. (11) reported that the incidence of implant failure for patients with an average age of 45.3 years was 36.3% without fusion. In our study, the incidence of screw breakage was 4% in group B. The reason for the lower incidence of screw breakage in our study might be that elderly patients are less active than young ones, which could also explain why our patients with screw breakage were asymptomatic.

The functional and radiological outcomes were similar between the two groups, except for the LMR. Kim et al. (20) reported that in final radiographs after implant removal, the mean motion angle of the fixed segment in the sagittal plane was 14.2° for patients with an average age of 28 years. Jeon et al. (7) reported that for patients who underwent short-segment fusion surgery with an average age of 39.7 years, the segmental motion angle was 1.6° ± 1.5° at the time of implant removal, and this increased significantly to 5.8° ± 3.9° at the 1-year follow-up visit. In our series, although statistical significance was found in LMR, the mean LMR only differed by 0.8° (group A 4.4°, group B 3.6°) between the two groups and 1.3° (final follow-up 4.4°, preimplant removal 3.1°) after implant removal in group A, which was questionable for the clinical significance. Mean TMR differed only by 1.2° with no significant difference between the two groups. In this study, the motion range of the waist in group A was lower than that in other studies, and this might be related to the lower waist activity of elderly patients. No neurological symptoms were found due to the degeneration of the adjacent segments at the latest follow-up.

Many studies have reported the correction loss of the kyphotic angle or vertebral body height after surgery. Palmisani et al. (21) showed that the correction loss of segmental kyphosis (Cobb A) and wedging deformity (Cobb B) were 5.1° and 3.7°, respectively, with a mean follow-up of 14.2 months for patients with an average age of 45 years. In a 10-year follow-up period, Kocanli et al. (22) reported that for patients with a mean age of 30.1 years, the correction loss of Cobb A and Cobb B were 8.06° and 3.49°, respectively. Chou et al. (11) reported that the correction loss of the kyphotic angle and vertebral body height was unrelated to implant removal in 69 patients with a mean age of 45.3 years after a mean follow-up of 66.2 months, and the correction loss of their AVHR was only 0.1%. In our series, the fracture levels we treated were from T11 to L4 and no hyperkyphosis was found during the entire follow-up. However, the correction loss of Cobb A, Cobb B, and AVHR were 7.8°, 4.1°, and 10.6%, respectively, and these were more serious than those reported in other studies. This could be attributed to the following reasons: (1) In our study, almost half of the patients had osteoporosis (T-score ≤−2.5) with a median T-score of −2.4 (−2.1 to −5.1). (2) All the patients underwent short-segment fixation. Atlay et al. (23) reported that the correction loss of sagittal angles and heights was more common and larger in short-segment fixation surgery than in long-segment fixation. However, the disadvantages of long-segment fixation (23) that includes sacrificing more motion segments, greater costs, prolonged surgical time, and higher complication rate, are also difficult to accept, especially for the elderly.

Tian et al. (24) concluded that percutaneous short-segment pedicle instrumentation in cases with satisfactory results could replace extensive open surgery in many cases and did not increase related complications. Lee et al. (25) reported that percutaneous fixation demonstrated favorable radiological and clinical outcomes for the treatment of unstable thoracolumbar and lumbar fractures compared to open Schanz screw stabilization. The results of our study were consistent with those of these studies. However, open surgery using Schanz screws for reduction has its own advantages. Compared to the open surgery group, the minimally invasive group showed better outcomes in terms of operation time, blood loss, and ODI within 6 months, with similar results in other functional and radiological outcomes.

Jor et al. (26) found that the hypotension rate after the induction of general anesthesia, that can increase postoperative morbidity and mortality, depends on age, degree of blood pressure decompensation prior surgery, and the presence of diabetes mellitus type II. Meanwhile, in our series, the proportion of elderly individuals with hypertension or diabetes was 38.5%. Consequently, a second surgery under general anesthesia would mean a higher risk for the elderly.

This study has some limitations. This was a single-center retrospective cohort study, and the number of patients included was small. In addition, the choice of implant removal was not randomized, and the removal time for each patient was not consistent. Hence, we recommend a multi-center prospective randomized controlled trial to confirm our findings. Although all the parameters were measured three times by the two authors with the arithmetic mean used, bias may still be found.

## Conclusion

We found similar radiological and functional outcomes in elderly patients, regardless of implant removal. Furthermore, the incidence of screw breakage was lower, and no obvious symptoms were found after breakage in elderly patients. Although the LMR increased after removal, no obvious benefits were observed for overall waist mobility. Additionally, the removal surgery would increase the medical expenses. Considering the risks of further surgery under general anesthesia for the elderly, removal of the implants may not be necessary. Nevertheless, patients should be informed about the possibility of implant breakage and accelerated degeneration of the adjacent segments before surgery.

## Data Availability

The original contributions presented in the study are included in the article/Suplementary Material, further inquiries can be directed to the corresponding author/s.
